# Correction: Representation of foreseeable choice outcomes in orbitofrontal cortex triplet-wise interactions

**DOI:** 10.1371/journal.pcbi.1009710

**Published:** 2021-12-28

**Authors:** Emili Balaguer-Ballester, Ramon Nogueira, Juan M. Abolafia, Ruben Moreno-Bote, Maria V. Sanchez-Vives

There was an error in the spelling of an author’s name; Juan M. Abofalia should be Juan M. Abolafia.

The was also an error in one of the grant numbers in the funding statement for this article. The funding statement should read as follows:

“This work was supported by EU H2020 Research and Innovation Programme under Grant Agreement No. 945539 (HBP SGA3), BFU2017-85048-R Spanish Ministry of Science to MVSV and by the project Async-Prop, Bournemouth University-IDIBAPS, RED11549, Partnering Project of the HBP SP3 (EU H2020 Research and Innovation Programme) to EB-B. The funders had no role in study design, data collection and analysis, decision to publish, or preparation of the manuscript.”
