# Supplementary figures and images for: Representation of foreseeable choice outcomes in orbitofrontal cortex triplet-wise interactions
Source: PLoS Comput Biol. 2020 Jun 24;16(6):e1007862. doi: 10.1371/journal.pcbi.1007862 (PMC7313741; doi:10.1371/journal.pcbi.1007862)

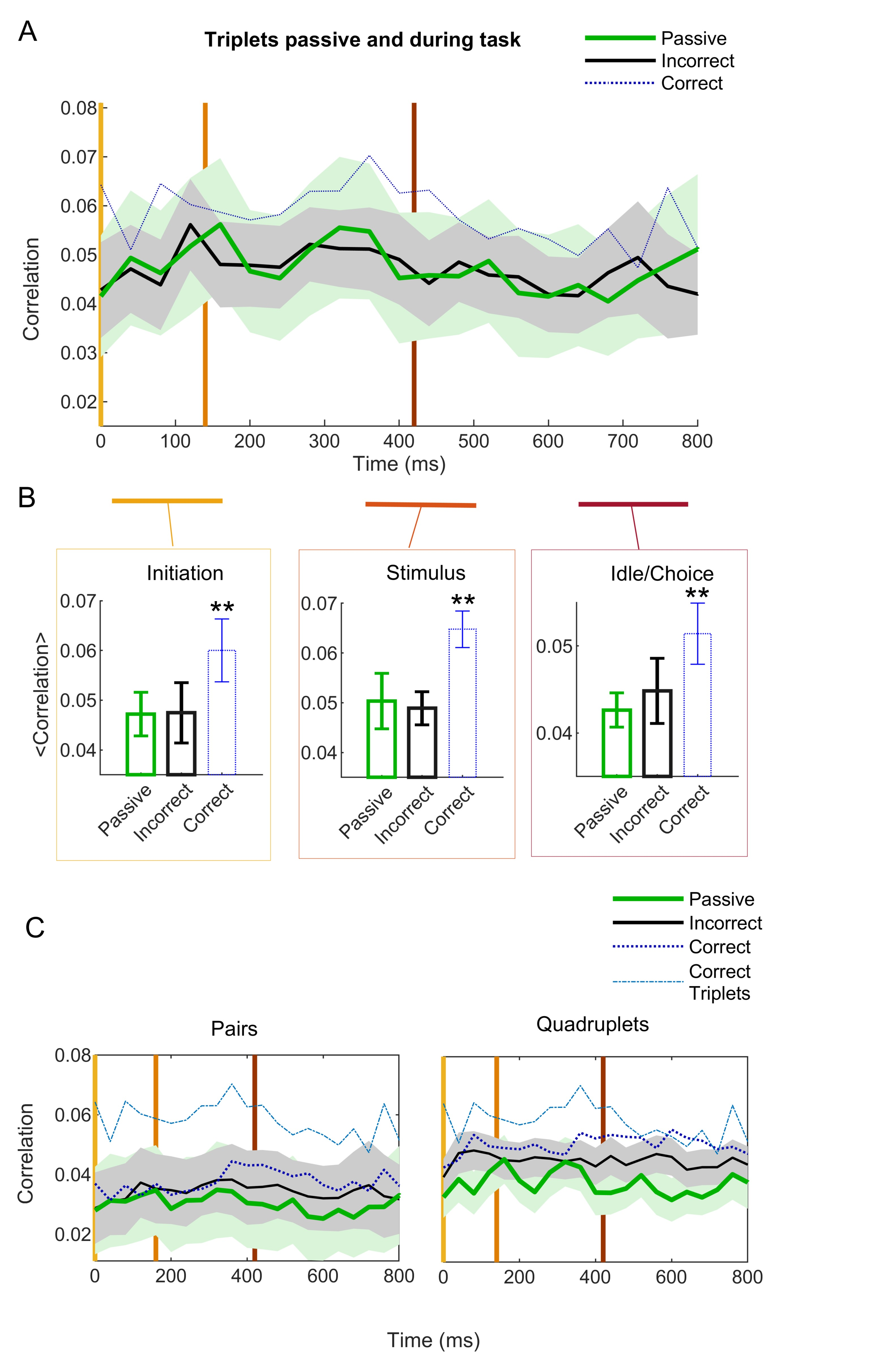

Supplement: S1 Fig — (A) Trial-averaged triple-wise correlations (Eq (1) in Materials and methods) further averaged across ensembles having n ≥ 5 units used in Fig 1. Correlations were computed during trials in which animals were passively exposed to the same set of stimuli as in Fig 1, but in which the reward was not delivered (green line). Vertical lines indicate the average position of different salient events (see Fig 1). (B) Same as in (A) during three periods of interest: trial initiation (left), stimulus offset (central) and choice (right) (Materials and methods), error bars are SD. Incorrect and passive trials and indistinguishable during the entire trial (passive vs incorrect T(40) = 0.344, p = 0.72, likewise for each one of the individual periods, data normal per Lilliefors test, p > 0.3); whilst correct and passive differ (T(40) = −6.59, p = 2.1 10−7 Bonferroni corrected; MANOVA for correct, passive and incorrect groups, Wilks’ ⋀ = 0.83 for correct versus passive subspace, p = 5.3 ⋅ 10−6). (C) Passive pairwise (left) and quadruplet-wise correlations (right) are shown for comparison. (TIF) [file pcbi.1007862.s001.tif]

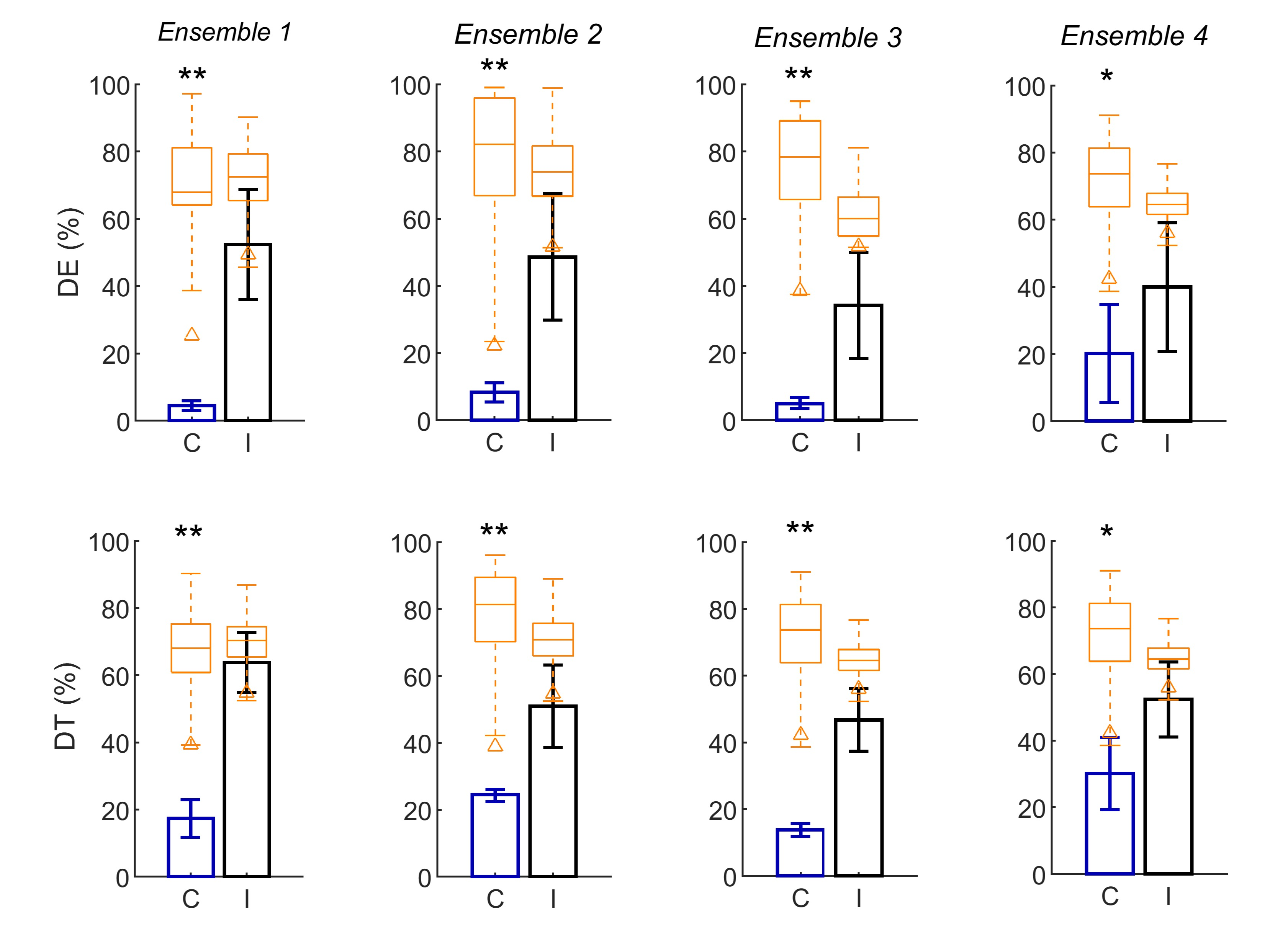

Supplement: S2 Fig — As in Figs 2–5, S3 Fig, an optimal regularized kernel-discriminant (order 3) was used to compute the mean of the six-fold-ahead causally cross-validated value of the decoding error (DE, top) and the trajectory divergence index (DT, bottom) for each ensemble. Blue bars show the index values for correct choices (“C”), black the average through the rest of choices (“I” stands for Incorrect choice). *p < 0.05, **p < 0.001, Wilcoxon rank sum tests. Orange boxplots show the quartiles, whiskers indicate outliers, triangle markers the 1% percentile of n = 1000 bootstraps drawn for the permutation tests, see Materials and methods. (TIF) [file pcbi.1007862.s002.tif]

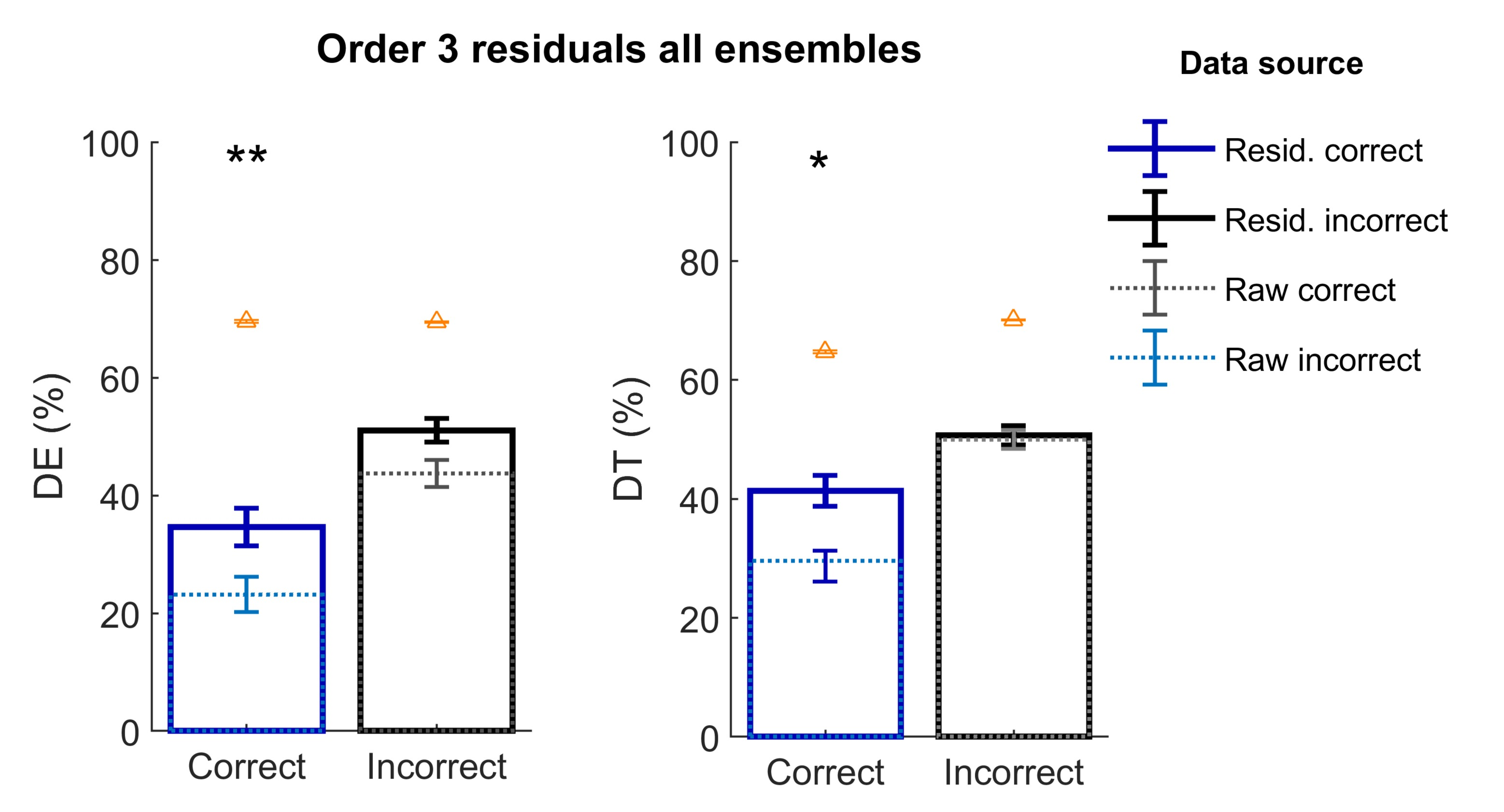

Supplement: S3 Fig — See Materials and methods, Eq (2) in the main text. As in Figs 2–5, S2 Fig, an optimal regularized kernel-discriminant (order 3) was used to compute the mean of the six-fold-ahead causally cross-validated value of the decoding error (DE) and the trajectory divergence index (DT) for all the ensembles recorded (n = 82 units). Blue bars show values for correct choices, black the average through the rest of choices, blue and black error bars are SEM. Dotted lines indicate decoding results for the original data for benchmark (Fig 4). Differences between correct and incorrect trials are significant both for DE (Wilcoxon rank sum, W = 19363, p = 3.9 ⋅ 10−5) and DT (W = 20695, p = 0.006). Orange markers show averages and SEM of n = 300 bootstraps drawn for the permutation tests of the residual data, see Materials and methods. (TIF) [file pcbi.1007862.s003.tif]

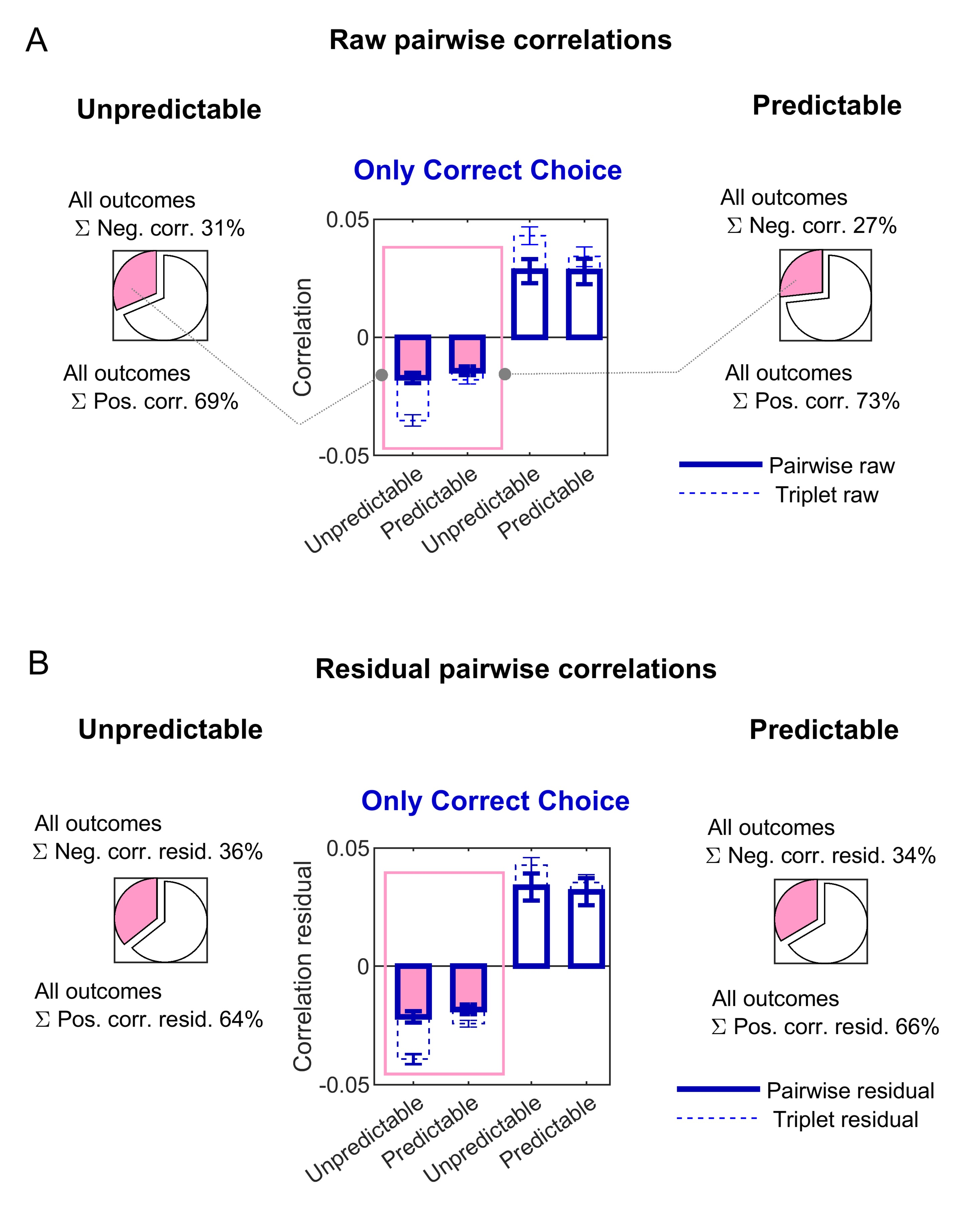

Supplement: S4 Fig — (A) Average correlations only for correct choices. Like in Figs 6C and 7C, left and right pie charts show the total fraction of positive and negative correlations for all ensembles and trials. Dashed lines indicate the mean triplet-wise correlations shown in Fig 6C for comparison. (B) The same analysis as in (A) but for partial pairwise correlations. See also Figs 6 and 7 for raw and partial triplet-wise correlations respectively. (TIF) [file pcbi.1007862.s004.tif]

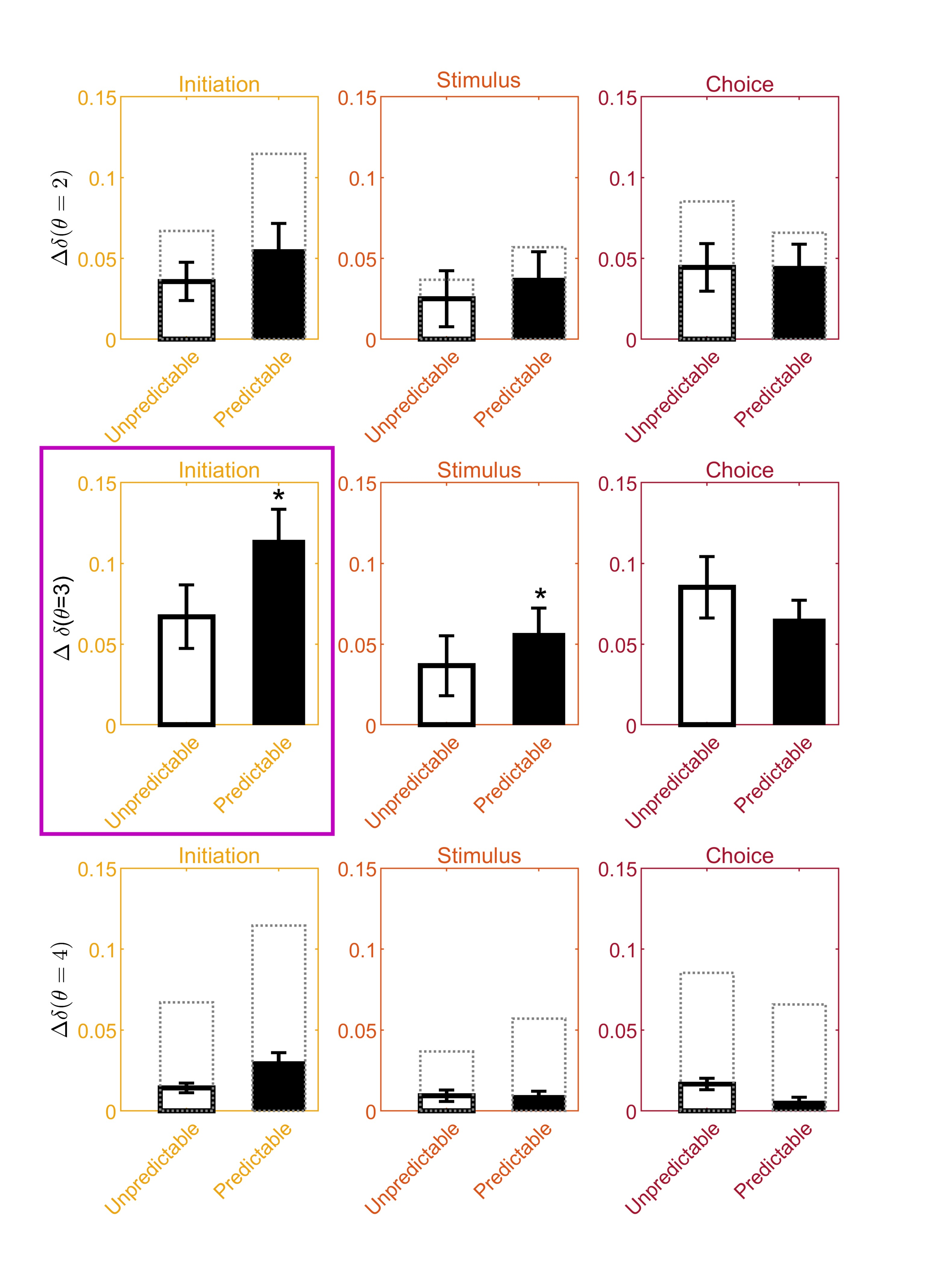

Supplement: S5 Fig — The jth differential correlation coefficient Δδpredictable(unpredictable)(j; θ) is the difference between positive (δ+) and negative (δ−) deltas; where δ+ (−) consist of the difference between positive (negative) correlations during correct and incorrect trials, aggregated during a specific time period (see Eq (S18)). The figure shows mean differential correlation coefficients for θ = 2 (top row), θ = 3 (middle row) and θ = 4 (bottom row), bars are SEM. Consistently with Fig 6 ((A) and (B), right panels), the mean Δδpredictable(θ = 3) is significantly stronger than Δδunpredictable(θ = 3) during early stages of the trial; especially before the upcoming stimulus becomes available (trial initiation period, left panel in middle row, Wilcoxon rank-sum W = 122311, p = 0.015, n = 359; stimulus period, p = 0.023). This effect does not reach significance neither for pairwise (top row) nor for quadruple-wise correlations (bottom row, dotted lines show the third order correlations, Δδ(θ = 3), for comparison). (TIF) [file pcbi.1007862.s005.tif]
