# Supplementary material for: Representation of foreseeable choice outcomes in orbitofrontal cortex triplet-wise interactions
Source: PLoS Comput Biol. 2020 Jun 24;16(6):e1007862. doi: 10.1371/journal.pcbi.1007862 (PMC7313741; doi:10.1371/journal.pcbi.1007862)
Supplement: S1 Methods — (PDF) [file pcbi.1007862.s006.pdf]

Supplementary Methods: Representation of Foreseeable Choice Outcomes in Orbitofrontal Cortex Triplet-wise Interactions

Emili Balaguer-Ballester[1,2*], Ramon Nogueira[3], Juan M. Abofalia[4], Ruben Moreno-Bote[5,6,7‡], Maria V. Sanchez-Vives[4,8‡]

1 Department of Computing and Informatics, Faculty of Science and Technology, Bournemouth University, Poole, UK.
2 Bernstein Center for Computational Neuroscience, Medical Faculty Mannheim and Heidelberg University, Mannheim, Germany.
3 Center for Theoretical Neuroscience, Mortimer B. Zuckerman Mind Brain Behavior Institute, Columbia University, New York, New York, USA.
4 IDIBAPS (Institut d'Investigacions Biomèdiques August Pi i Sunyer), Barcelona, Spain.
5 Department of Information and Communication Technologies, Universitat Pompeu Fabra, Barcelona, Spain.
6 Center for Brain and Cognition, Mercé Rodoreda building (Ciutadella campus), Barcelona, Spain.
7 Serra Húnter Fellow Programme, Universitat Pompeu Fabra, Barcelona, Spain.
8 ICREA (Institució Catalana de Recerca i Estudis Avançats), Barcelona, Spain.

‡These authors contributed equally to this work.
∗ eb-ballester@bournemouth.ac.uk

Methodology and Implementation

This section provides additional details on the implementation of the decoding algorithm. Most of this material has been discussed elsewhere [1–7] and is shown here for clarity.

Classical Fisher discriminant analysis

The optimization program of a standard non-regularized Fisher discriminant provides a $(c-1)$-dimensional subspace embedded in an n-dimensional firing-rate space of $l = \sum_{y=1}^{c} l_y$ vectors $\mathbf{x}(t)$ of dimension $(n \times 1)$, where t is the time bin during a trial, z encodes the behavioral category (the type of response in the trial), c is the number of different behavioral responses and l_y is the total number of vectors per response category y in a given dataset (Materials and Methods, Decoding algorithm section). The $c-1$ subspace axes $\mathbf{w}$ $(n \times 1)$ are chosen to maximize the between-categories variance whilst minimizing the within-category variance of the projected rate vectors onto the subspace; that is, to maximize the Rayleigh coefficient [7]:

$$\mathbf{w} : max_{\mathbf{w}} J(\mathbf{w}) = \frac{\mathbf{w}^T \tilde{M} \mathbf{w}}{\mathbf{w}^T \tilde{N} \mathbf{w}}, \tag{S1}$$

in which the between-category (*between-class* [7]) variance matrix $\tilde{M}$ $(n \times n)$ is,

$$\tilde{M} = \sum_{y=1}^{c} (\tilde{\boldsymbol{\mu}}_y - \tilde{\mathbf{m}}) \cdot (\tilde{\boldsymbol{\mu}}_y - \tilde{\mathbf{m}})^T, \; \tilde{\mathbf{m}} = \frac{1}{c} \cdot \sum_{y=1}^{c} \tilde{\boldsymbol{\mu}}_y, \tag{S2}$$

$\tilde{\boldsymbol{\mu}}_{y=y_0} = \frac{1}{l_{y_0}} \cdot \sum_t \mathbf{x}(t)$ is the $(n \times 1)$ mean vector during all trials in a given dataset in which the behavioural response category is y_0; that is, the sum runs $\forall \mathbf{x}(t)$ such that $y(\mathbf{x}(t)) = y_0 \in \{1, ..., c\}$. Likewise, the $(n \times n)$ within-category (*within-class* [7]) covariance is defined as:

$$\tilde{N} = \sum_{y=1}^{c} \sum_{t=t_1}^{t_{l_y}} (\mathbf{x}(t) - \tilde{\boldsymbol{\mu}}_y) \cdot (\mathbf{x}(t) - \tilde{\boldsymbol{\mu}}_y)^T \,. \tag{S3}$$

The discriminant program (Eq (S1)) can be straightforwardly solved for instance by Lagrange multipliers or by computing the maximum of the Rayleigh quotient $J(\mathbf{x})$, the latter renders the following generalized eigenvalue problem for the optimal discriminant directions $\mathbf{w}$ [8],

$$\tilde{M}\mathbf{w} = (\tilde{N} + \tilde{\lambda} \cdot \mathbb{I})\mathbf{w}, \tag{S4}$$

where $\mathbb{I}$ is the $(n \times n)$ identity matrix and $\tilde{\lambda}$ is an arbitrary regularization constant to avoid over-fitting [9]. The subspace spanned by the $c - 1$ eigenvectors $\mathbf{w}$ is computed from observations $\mathbf{x}(t)$ corresponding to the block of 40 trials (termed B_k in the Reliability over trials analysis section, Materials and Methods) and then a new firing-rate observation $\mathbf{x}(t)$ in a future block B_{k+1} is projected onto each one of the optimal subspace axes previously determined, $f(t) = \mathbf{w}^T \cdot \mathbf{x}(t)$.

The $(1 \times (c - 1))$ vector $\mathbf{f}(t)$ contains the projection of the observation $\mathbf{x}(t)$ onto each discriminant subspace dimension. This projection facilitates setting a threshold for decoding, since observations corresponding to distinct categories are optimally separated in the discriminant subspace. If the distribution of $\mathbf{f}(t)$ for all the observations in a block is multivariate normal, the predicted category $y(\mathbf{x}(t)) = y_0$ is the one rendering the highest classification probability (Eq (7)), see Regularization and probabilistic interpretation in Materials and Methods), where mean and class-covariances were estimated from a previous dataset B_k (Materials and Methods, Reliability over trials analysis).

This Fisher discriminant criterion (Eq (S1)) is equivalent to a linear discriminant for normal within-category probabilities in the state space of firing-vectors $\mathbf{x}(t)$ with equal covariances (quadratic discriminant if covariances differ) [7]. This can be easily checked for two classes by differentiating the log-posteriors (Eq (7)) [8]. In this setting, it is Bayes-optimal and a superior choice over other classifiers [9]. In addition, it provides a low-dimensional visualization of the optimal subspace spanned by the eigenvectors $\mathbf{w}$ (when conveniently orthogonalized).

Moreover, it is well-known that the discriminant optimization criterion (Eq (S1)) can be equivalently reformulated as a ridge-like regression problem [7],

$$\mathbf{w} : \; min_{\mathbf{w},\beta} \, (\tilde{\xi}(\mathbf{w}, \beta)^2 + \tilde{\lambda} \cdot \mathbf{w}^T \cdot \mathbf{w}),$$

where

$$\tilde{\xi}(\mathbf{w}, \beta)^2 = \sum_t (\hat{f}(t) - y(\mathbf{x}(t)))^2, \text{ in which } \hat{f}(t) = \mathbf{w}^T \cdot \mathbf{x}(t) + \beta \,. \tag{S5}$$

As shown in [7], the optimal discriminant direction $\mathbf{w}$ obtained with this approach coincides with the first sorted eigenvector of (S4). This least-squares formulation often allows for more transparent and efficient solutions, as will be discussed below in the context of the kernel discriminant.

Regularized kernel-Fisher discriminant

However, for enlarged states spaces incorporating unit interactions (Eq (8), section State spaces and correlations) the substitution $\mathbf{x}(t) \rightarrow \boldsymbol{\phi}(\theta, \mathbf{x}(t))$ for $\theta > 1$ may render the discriminant optimization program computationally unfeasible regardless how it is reformulated, since vectors $\boldsymbol{\phi}$ are high-dimensional.

This problem can be circumvented leveraging the fact that only products among of such high-dimensional vectors are used in the Rayleigh quotient (Eq (S1)), whilst there is no need for an explicit representation of the vectors $\boldsymbol{\phi}(\theta, \mathbf{x}(t))$ or highly sparse covariance matrices $\tilde{M}$, $\tilde{N}$ [3]. Vector products in the state space used here are provided by Eq (5)) in Materials and Methods, this operation is well-known as the kernel-trick (Eq (9)) [1]. Thus, the projected data for the observation $\mathbf{x}(t)$ onto each one of the discriminant subspace axes $\mathbf{w}$ can be equivalently computed as [3],

$$f(t) = \mathbf{w}^T \cdot \boldsymbol{\phi}(\theta, \mathbf{x}(t)) = \sum_{t'=t_1}^{t_l} \alpha_{t'} \cdot \boldsymbol{\phi}(\theta, \mathbf{x}(t'))^T \cdot \boldsymbol{\phi}(\theta, \mathbf{x}(t)), \tag{S6}$$

where l is the number of observations and the high-dimensional vector $\mathbf{w}$ has been expanded in $\boldsymbol{\phi}$ vectors using $\alpha_{t'}$ coefficients, which are the ones to be determined [1]. Equivalently,

$$f(t) = \sum_{t'=t_1}^{t_l} \alpha_{t'} \cdot k(\mathbf{x}(t), \mathbf{x}(t')), \text{i.e., } F_j = K\boldsymbol{\alpha}, \tag{S7}$$

where $\boldsymbol{\alpha}$ is a $(l \times 1)$ vector of coefficients, K is the $l \times l$ symmetric gram matrix of entries provided by the kernel function $k(\mathbf{x}(t), \mathbf{x}(t'))$ (Eq (5)) and $f(t)$ are entries of a $(l \times (c-1))$ matrix F. This expansion can be obtained for successive eigenvectors $\mathbf{w}$. In short, $f(t)$ are precisely the entries of the output matrix F (Eq (6) in Materials and Methods), and F_j are its $j = 1, ..., c-1$ column vectors.

Likewise, the Rayleigh quotient (Eq (S1)) depends only on products of high-dimensional vectors and thus can be fully expressed in terms of gram matrices operations. Thus, straightforward algebra using the kernel trick enabled [3] to recast Eq (S1) as:

$$\boldsymbol{\alpha} : \; max_{\boldsymbol{\alpha}} \frac{\boldsymbol{\alpha}^T M \boldsymbol{\alpha}}{\boldsymbol{\alpha}^T N \boldsymbol{\alpha}}, \tag{S8}$$

where the $(l \times l)$ matrices M and N are described below. The problem has been now parsed into smaller and denser matrices and the computation of $\boldsymbol{\alpha}$ becomes feasible. The matrix M is defined as,

$$M = \sum_{y=1}^{c} (\boldsymbol{\mu}_y - \mathbf{m}) \cdot (\boldsymbol{\mu}_y - \mathbf{m})^T, \; \mathbf{m} = \frac{1}{c} \cdot \sum_{y=1}^{c} \boldsymbol{\mu}_y, \tag{S9}$$

where $\boldsymbol{\mu}_{y=y_0} = \frac{1}{l_{y_0}} \cdot K \mathbf{1}_{y_0}$ and $\mathbf{1}_{y_0}$ is a $(l \times 1)$ vector of ones if each observation $\mathbf{x}(t)$ occur during a trial in which the behavioural response is y_0 and zero otherwise. In short, $\boldsymbol{\mu}_{y_0}$ is the mean of high-dimensional vector products during trials belonging to the category $y = y_0$.

Likewise, the matrix N can be easily derived as shown in [3],

$$N = K \cdot K^T - \sum_{y=1}^{c} (l_y \cdot \boldsymbol{\mu}_y \cdot \boldsymbol{\mu}_y^T), \tag{S10}$$

where K is again the gram matrix of entries provided in Eq (5). Thus, the generalized eigenvalue problem (S4) is recast as:

$$M\boldsymbol{\alpha} = \nu \cdot (N + \lambda \cdot K)\boldsymbol{\alpha}, \tag{S11}$$

which provides $c-1$ nonzero eigenvalues ν for all the ensembles analyzed, except for a small one (as further discussed below). Eigenvectors $\boldsymbol{\alpha}$ are then sorted in a descending eigenvalue order and Eq ((S7)) provides the projected data to the optimal subspace (further orthogonalized only for visualization on Figs 3 and 4 as discussed in Reliability over trials analysis, Materials and Methods).

Finally, like in the classical discriminant, the eigenvalue problem (Eq (S8)) shows parallels with a ridge regression (Eq (S5)). The matrix M is rank one by definition (Eq (S9)), in addition, only the direction of $\boldsymbol{\alpha}$ matters i.e., any multiple of $\boldsymbol{\alpha}$ is a solution of the eigenvalue problem (Eq (S8)). Thus, the numerator can be fixed to any value as a constraint, and a solution of Eq (S8) can be obtained from $\boldsymbol{\alpha} : min_{\boldsymbol{\alpha}} \left(\boldsymbol{\alpha}^T N \boldsymbol{\alpha} + \lambda \cdot \boldsymbol{\alpha}^T K \boldsymbol{\alpha}\right)$, which is precisely Eq (3) in Materials and Methods. This analogy with the ridge regression (Eq (S5)) can be brought further as demonstrated in [2] by rewriting the eigenvalue problem (Eq (S8)) as,

$$\boldsymbol{\alpha} : \; min_{\boldsymbol{\alpha},\beta} \left(\|\boldsymbol{\xi}(\boldsymbol{\alpha},\beta)\|^2 + \lambda \cdot \boldsymbol{\alpha}^T K \boldsymbol{\alpha}\right),$$

subject to:

$$\mathbf{1}_y^T \boldsymbol{\xi} = \mathbf{0}, \; \forall y \in \{1, ..., c\}, \tag{S12}$$

where the first summand is the $L2$ loss function, which here is the squared norm of the error vector $\boldsymbol{\xi}$ defined as $\boldsymbol{\xi} = K\boldsymbol{\alpha} + \beta \cdot \mathbf{1} - \mathbf{y}$. The symbol $\mathbf{1}$ is a $(l \times 1)$ vector of ones and the $(l \times 1)$ vector $\mathbf{y}$ contains the categories of the l observations $y(\mathbf{x}(t))$. Like for the classical discriminant, it was shown in [2] that the optimal least-squares solution $\boldsymbol{\alpha}$ is collinear with the dominant eigenvalue of Eq (S11).

The projected data F is typically normal, this is not surprising, since the $c-1$ axes of the discriminant subspace are combinations of $D \sim O(10^3)$ low-correlated dimensions (mean correlations < 0.07 in Figs 1-3, 6, 7, S1 Fig, and S4 Fig), approaching the conditions of the central limit theorem [8] and thus the largest Gaussian posterior provides the category prediction (Eq (7)).

Implementation considerations

To ensure that the penalization is effective enough to solve Eq (S8), the regularization constant λ was scaled by the grand average of the kernel matrix, $\frac{\mathbf{1}^T K \mathbf{1}}{l^2}$, where $\mathbf{1}$ is a $l \times 1$ vector of ones [6]. However, in one of the smallest ensembles (5 units, Fig 3) the generalized eigenvalue in Eq (S11) is still not computationally solvable and hence the predicted class cannot be computed. In this case, to reduce the sparsity in the matrix K, the low-pass filtered local field potential (LFP) was incorporated as an additional variable. The LFP was downsampled to 250 Hz to filter out the typical spiking activity component, and hence to provide non-overlapping information with respect to the units activity within the ensemble [10].

The computational cost of the optimization program is $O(l^3)$ per ensemble. With these considerations, $\boldsymbol{\alpha}$ is computable in standard hardware for data analytics (16-core cluster, Matlab parallel toolbox 2018, Matworks inc.) for the datasets of this study. However, for $l \times l$ matrices $l \gtrsim 10^4$ the computation is often inaccurate, specially for $\theta \geq 3$ polynomial state spaces or similar Hilbert spaces endowed with other kernels. Thus, even though not compulsory here, we recommend to use the formulation shown in Eq (S12) for *in vivo* recordings. This program can be approximately solved by least squares, which alleviates the imprecision typically associated with solving the

generalized eigenvalue problem (Eq (S11)). Besides the computational convenience, it is also more transparent for its equivalence with a ridge regression with constrains [2].

State-space Vectors and Correlations

Vectors in a θth-order state space (Eq (8)) can be trivially identified as follows: the product of any two vectors $\boldsymbol{\phi}(\theta, \mathbf{x}(t))^T, \boldsymbol{\phi}(\theta, \mathbf{x}(t'))$ in the high-dimensional, feature state space is provided by the kernel function,

$$\boldsymbol{\phi}(\theta, \mathbf{x}(t))^T \cdot \boldsymbol{\phi}(\theta, \mathbf{x}(t')) = k(\mathbf{x}(t, \mathbf{x}(t')) = (1 + \mathbf{x}(t)^T \cdot \mathbf{x}(t'))^{\theta} - 1, \tag{S13}$$

where the last term is simply a multinomial expansion of $n+1$ terms. Other popular kernel choices include the well-known Gaussian kernel, which is also universal (it can approximate an arbitrary continuous target function uniformly from a subset of the input state space) [11]. More specialized, spike train kernels such as the Schoenberg kernel, are designed to compute the similarity between pairs of spike trains without the need for binning [12]. However, neither of these kernels allow to establish a simple link with θ-order neuronal correlations, and hence our choice was the multinomial kernel operating on the firing rates (Eq (S13)), estimated in the smallest possible bins (see Electrophysiology section in Materials and Methods).

Thus, for $\theta = 2$, trivially expanding the binomial:

$$\begin{aligned}(1 + \mathbf{x}(t)^T \cdot \mathbf{x}(t'))^{\theta=2} - 1 = (1 + x_1(t) \cdot x_1(t') + x_2(t) \cdot x_2(t') + ... + x_n(t) \cdot x_n(t'))^2 - 1 \\ ... \\ = 2 \cdot x_1(t) \cdot x_1(t') + ... + 2 \cdot x_n(t) \cdot x_n(t') + 2 \cdot x_1(t) \cdot x_1(t') \cdot x_2(t) \cdot x_2(t') \\ +... + 2 \cdot x_1(t) \cdot x_1(t') \cdot x_n(t) \cdot x_n(t') + ... + 2 \cdot x_{n-1}(t) \cdot x_{n-1}(t') \cdot x_n(t) \cdot x_n(t') \\ +... + x_1(t)^2 \cdot x_1(t')^2 + ... + x_n(t)^2 \cdot x_n(t')^2 \,.\end{aligned} \tag{S14}$$

Simply comparing Eq (S14) with Eq (8) in Materials and Methods renders,

$$\begin{aligned}\boldsymbol{\phi}(\theta = 2, \mathbf{x}(t)) = [\sqrt{2} \cdot x_1(t), ..., \sqrt{2} \cdot x_n(t), ..., \sqrt{2} \cdot x_1(t) \cdot x_2(t), ..., \sqrt{2} \cdot x_1(t) \cdot x_n(t), \\ ..., x_1(t)^2, ..., x_n(t)^2)]^T = \\ [\sqrt{\binom{\theta = 2}{i_0 = 1, i_1 = 1, i_2, ..., i_n = 0}} \cdot x_1(t)^{i_1}, \\ \sqrt{\binom{2}{i_0 = 0, i_1, i_2 = 1, i_3, ..., i_n = 0}} \cdot x_1(t)^{i_1} \cdot x_2(t)^{i_2}, \\ ..., \\ \sqrt{\binom{2}{i_0, ..., i_{n-1} = 0, i_n = 2}} \cdot x_n(t)^{i_n}]^T \,.\end{aligned} \tag{S15}$$

Thus, the constraints (Eq (8)) on the $n+1$ binomial indexes are $i_0 < 2$, and $\sum_{k=0}^{n} i_k = 2$. Likewise, for an arbitrary θ (showing only a few terms for illustration of the well-known expansion):

$$(1+\mathbf{x}(t)^T\cdot\mathbf{x}(t'))^\theta-1=\binom{\theta}{i_0=\theta-1,i_1=1,i_2...,i_n=0}\cdot x_1(t)\cdot x_1(t')$$
$$+...+\binom{\theta}{i_0=\theta-1,i_1,...,i_{n-1}=0,i_n=1}\cdot x_n(t)\cdot x_n(t')$$
$$+...+\binom{\theta}{i_0=\theta-2,i_1,i_2=1,i_3,...,i_n=0}\cdot x_1(t)\cdot x_1(t')\cdot x_2(t)\cdot x_2(t') \quad \text{(S16)}$$
$$...$$
$$+...+\binom{\theta}{i_0,...,i_{n-1}=0,i_n=\theta}\cdot x_n(t)^\theta\cdot x_n(t')^\theta\,.$$

Like in Eq (S15), the coefficient of each jth component of the vector $\boldsymbol{\phi}(\theta,\mathbf{x}(t))$ is just the square root of the corresponding multinomial term (Materials and Methods, Eq (8)). Thus, after applying the normalization shown in Eq (11) in Materials and Methods, the $\theta^{th} \leq n$-order correlation between up to θ units $a_1,...,a_\theta$ within a n-units ensemble can be expressed as,

$$Corr(x_1,x_2,...,x_n;\theta)(t)=\frac{\sum_T\{\tilde{\boldsymbol{\phi}}(\theta,\mathbf{x}(t,T))\}_j}{(\sum_T\{\tilde{\boldsymbol{\phi}}(\theta,\mathbf{x}(t,T))\}_{p_1})^{\frac{1}{\theta}}\cdots(\sum_T\{\tilde{\boldsymbol{\phi}}(\theta,\mathbf{x}(t,T))\}_{p_n})^{\frac{1}{\theta}}}, \quad \text{(S17a)}$$
$$\text{where}\sum_T x_{a_1}(t,T)=...=\sum_T x_{a_\theta}(t,T)=0\,.$$

The vector components j and p_k, $k\in\{1,...,n\}$ refer to a specific combination of values for the indexes $i_{a_1},...,i_{a_\theta}\geq 0$ and zero for the remaining indexes, which are subject to the constraints indicated in Eq (8); that is,

$$j=j(i_{a_1},...,i_{a_\theta}) := \begin{cases} i_k\geq 0, & \text{if } k\in\{a_1,...,a_\theta\}\\ i_k=0, & \text{otherwise.}\end{cases}$$
$$p_k=p_k(i_{a_1},...,i_{a_\theta}) := \begin{cases} i_k=\theta, & \text{if } k\in\{a_1,...,a_\theta\}\\ i_k=0, & \text{otherwise.}\end{cases} \quad \text{(S17b)}$$
$$0\leq i_0<\theta,\ 0\leq i_{k\neq 0}\leq\theta,\ \sum_{k=0}^{n} i_k=\theta\,.$$

Finally, like in Eq (11) in Materials and Methods, the trial-average correlation for a fixed-choice outcome category and for all n units in an ensemble (shown in Figs 1-3, 6, 7, S1 Fig and S4 Fig) is simply the average of Eq (S17a) for all possible θ-order coefficients, $\binom{n+\theta-1}{n-1}-n$.

Differential correlation

We computed an auxiliary index to visualize differences between positive and negative correlations during different trial stages and types, that we term the *differential* correlation coefficient $\Delta\delta$. For each $\theta-$order j^{th} correlation coefficient among $n>\theta$ units (see equations (S17a) and (S17b)), we calculate:

$$\Delta\delta_{predictable\ (unpredictable)}(j;\theta)=\delta^{+}_{predictable\ (unpredictable)}(j;\theta)-\delta^{-}_{predictable(unpredictable)}(j;\theta), \quad \text{(S18)}$$

where $\delta^{+(-)}_{predictable\ (unpredictable)}$ is termed here the *delta* positive (negative) correlation coefficient during one of the time periods (initiation, stimulus, choice). Focusing on positive correlations (+) and on trials that follow an unsuccessful outcome (predictable), $\delta^{+}_{predictable}$ is simply defined as:

$$\delta^{+}_{predictable}(j;\theta) := / \sum_{t=t_0}^{t_1} Corr(x_1, x_2, ..., x_n; \theta)^{+}_{correct}(t) - Corr(x_1, x_2, ..., x_n; \theta)^{+}_{incorrect}(t)/, \quad \text{(S19)}$$

where both correct and incorrect trial are selected after an incorrect choice (that is, predictable) and + refer to positive $\theta - order$ correlation coefficients. The same quantity is computed for negative correlations (−) and unpredictable trials, resulting in the two values ($\Delta\delta_{predictable}$, $\Delta\delta_{unpredictable}$) shown in S5 Fig. In short, $\Delta\delta_{predictable\ (unpredictable)} > 0$ suggest that positive correlations express more strongly the difference between correct and incorrect trials than negative ones.

References

1. Schölkopf B, Smola A. Learning with Kernels: Support Vector Machines, Regularization, Optimization, and Beyond. Adaptive Computation and Machine Learning. Cambridge, MA, USA: MIT Press; 2002.

2. Mika S, Ratsch G, Müller K. A mathematical programming approach to the kernel fisher algorithm. In: Neural Information Processing Systems. vol. 13. MIT press; 2001. p. 570–576.

3. Mika S, Rätsch G, Weston J, Schölkopf B, Müller KR. Fisher discriminant analysis with kernels. In: Proceedings of the 1999 IEEE Signal Processing Society Workshop. vol. 9. Max-Planck-Gesellschaft. IEEE; 1999. p. 41–48.

4. Balaguer-Ballester E, Lapish C, Seamans J, Durstewitz D. Attracting dynamics of frontal cortex ensembles during memory guided decision making. PLoS Computational Biology. 2011;7:e1002057. EB–B and CCL contributed equally. doi:10.1371/journal.pcbi.1002057.

5. Balaguer-Ballester E, Tabas-Diaz A, Budka M. Can We Identify Non-Stationary Dynamics of Trial-to-Trial Variability? PLoS ONE. 2014;9(4):1–13. doi:10.1371/journal.pone.0095648.

6. Lapish CC, Balaguer-Ballester E, Seamans JK, Phillips AG, Durstewitz D. Amphetamine Exerts Dose-Dependent Changes in Prefrontal Cortex Attractor Dynamics during Working Memory. Journal of Neuroscience. 2015;35(28):10172–10187. EB–B and CCL contributed equally. doi:10.1523/JNEUROSCI.2421-14.2015.

7. Duda RO, Hart PE, Stork DG. Pattern Classification. 2nd ed. New York: Wiley; 2001.

8. Bishop C. Pattern recognition and machine learning. New York: Springer-Verlag; 2007.

9. Hastie T, Tibshirani R, Friedman J. The elements of statistical learning: data mining, inference and prediction. 2nd ed. New York: Springer; 2009.

10. Capone C, Mattia M, Del Giudice P, Rebollo B, Sanchez-Vives MV, Muñoz A, et al. Slow Waves in Cortical Slices: How Spontaneous Activity is Shaped by Laminar Structure. Cerebral Cortex. 2017;29(1):319–335. doi:10.1093/cercor/bhx326.

11. Micchelli CA, Xu Y, Zhang H. Universal Kernels. Journal of Machine Learning Research. 2006;7:2651–2667.

12. Park IM, Seth S, Paiva ARC, Li L, Principe JC. Kernel Methods on Spike Train Space for Neuroscience: A Tutorial. IEEE Signal Processing Magazine. 2013;30(4):149–160. doi:10.1109/MSP.2013.2251072.
