# Supplementary material for: Representation of foreseeable choice outcomes in orbitofrontal cortex triplet-wise interactions
Source: PLoS Comput Biol. 2020 Jun 24;16(6):e1007862. doi: 10.1371/journal.pcbi.1007862 (PMC7313741; doi:10.1371/journal.pcbi.1007862)
Supplement: Attachment — Submitted filename: Reviewers_Responses_Letter_Balaguer_Ballester_et_al.pdf [file pcbi.1007862.s007.pdf]

Our manuscript is driven by the need to understand the patterns of shared variability in orbitofrontal cortex ensembles underlying the predictability of the outcome. This has generated a spectrum of responses from the two reviewers, each with a different focus on the many components of the manuscript. We are delighted to address each in turn.

Reviewer 1

The detailed analysis of the reviewer on the manuscript and specifically on the novelty and limitations of this approach is most welcome. The reviewer homes in on some potential weaknesses in the manuscript, especially on the insight provided by the mapping between neuronal interactions and choice outcome. Please find below our specific replies and the main changes highlighted in the revised version.

I. General concerns:

Reviewer comment 1 (RC1): *"This manuscript follows up on earlier work by the authors on population coding in the rodent frontal cortex, and specifically refers to their experimental design involving rat behavior on an interval discrimination task.*

The central finding here, which differentiates this manuscript from earlier ones, is that triplet correlations are higher in correct than incorrect trials for the duration of the trial."

The reviewer's opening comment points out to the use of a methodology, part of it previously tuned in a different frontal region and task, for ensemble decoding in OFC. We concur with the reviewer that we fell short in clarifying the novel components of our results. This will be addressed in the next queries (***RC2, RC3***).

In addition, we did not stress two methodological details specifically designed for this setting. As the reviewer indicates, we resorted to the kernelized approach as in our previous studies in ACC [e.g., refs. 37, 55 in the main manuscript]. However, it is worth noting that the explicit mapping between theta-order person correlations and this reproducing-kernel Hilbert space [equation (11) and specially the general case, equations (S17a), (S17b)] is, to our knowledge, novel not just in this application but more broadly in the kernel literature (see also ***RC2*** below).

Less salient, the alternative formulation of the decoder (equation (S12)) differs from previous works. This recast of the algorithm is not novel in machine learning [Supplementary Material, ref. 2]. However, specifically for *in vivo* recordings, we propose here that it can prevent rounding errors when inverting large kernel matrices of firing rates. This broadens the scope of this approach beyond our previous studies, limited by the dataset size, as is typically the case in kernel machines [ref. 82].

We have clarified this succinctly in the Supplementary material, lines 123-125 and in the main manuscript lines 154, 617-619. The key result of this manuscript will be addressed in detail in ***RC3***.

Reviewer comment 2 (RC2): *"I have a number of concerns which dampen my confidence in the findings and their (apparent over-)interpretation. The central result is: because on trials that follow an incorrect one, the stimulus is deterministic and equal to the one in the previous trial, the animal's response is often correct --presumably because the deterministic answer can be fetched from working memory--, and something is different in population activity in the OFC. So, OFC activity contains an "I did it wrong last time, so the next stimulus is like the last one" signal which correlates with correct performance in the present trial. But this finding (which I do buy -- see below) is not especially new: the present analysis was unnecessary to find it, and it's been reported before. The present work does not clarify the signal's origin or whether it is read or readable by other brain areas.*

So the point of this manuscript is then to individuate the substrates of the signal, in terms of features of neuronal activity. To this end, the authors perform a series of analyses, but their value is unclear based on the manuscript in its present version."

Thank you for raising this general concern, which refers to a fundamental aspect that we didn't convey sharply enough. This issue is addressed in ***RC3*** below using new analyses, whilst here we just provide a brief clarification.

- As the reviewer indicates, we previously showed that single units in rodent OFC encode variables that can potentially enable the prediction of the upcoming choice [ref. 40]. As a result, it is possible to decode the choice, yet with suboptimal accuracy (compare and order=1 and the other orders in the present manuscript, Fig. 4, top panels).

  However, this was the case irrespective of whether the outcome is predictable [Fig. 6 in ref. 40] or not [Fig. 8 in ref. 40]; and it was thus unclear if/how the decoding in OFC depends on consistent successes beyond the immediately previous trial.

  In contrast, we hypothesise here that decoding of OFC signals is predictability- and cumulative success-dependent (Fig 5B, right panel), leveraging the discriminative capacity of higher-order interactions. We do agree with the reviewer that is an overstatement to assume causality of correlations in task processing; and discuss this further in ***RC3*** below.

  It is worth clarifying that the specific mechanism underlying decoding we propose is that infrequent anti-correlation during the early stages of the trial hinders decoding capacity only for unpredictable choice outcome trials (Fig 6A right, and 6C central panel). This wasn't proposed or tested previously in the literature, to our best knowledge. We discuss this in more detail and indicate the edited lines in ***RC3***.

- It would be convincing to evaluate this mechanism in areas connected with OFC and demonstrate causality using Granger analysis for instance. We discuss this succinctly in the main manuscript lines 373-375 and now we added lines 404-405. In addition, on lines 398-404, we hypothesise that the task-space representation for two consecutive trials in OFC units found in ref. [40] could be tuned by the mechanism proposed here operating at a longer time scale to optimize decoding, but the downstream route of this mechanism is unknown.

Reviewer comment 3 (RC3): *"The problematic issues begin already with the introduction. Lines 89-93: "three-way correlations are systematically involved in successful task-processing". Are the correlations really processing the task? In the light of everything that comes later, isn't it that the signal has something to do with a memory of the previous trial's history, and thus presumably a state change or working memory trace? There is no evidence in the paper that correlations carry out "processing" independent of trial history to lead to a correct outcome. Any difference between correct and incorrect trials is present in the figures from the outset of the trial, as the authors themselves say, so the only way it can be explained is as a state- or memory-dependent effect."*

"[…] not a convincing way to demonstrate that the effect is exclusively down to the triplet interactions".

Thank you, we agree with the reviewer in that we are not demonstrating that triplet-wise 'noise' correlation coefficients have a causal influence in the decision. Thus, to assume a processing in a causal sense is an overstatement, and this aspect is toned down in the revised version: we indicate instead that correlations are associated with the task outcomes/furnish the state space representation for decoding (visualized in Fig 3B); see previous text and multiple edits in the main manuscript, e.g., in lines 1 (title), 91-92, 114, 117-118, 142, 339, 357, 384-386 and other edits discussed below in this letter.

However, to further clarify our angle, we have also added the following three new analyses (new figures S1, S3, S5).

- **New dataset and new analysis 1 (new Supplementary Figure S1)**

  As a first extra piece of support, we incorporated a new dataset (small ensembles of this dataset were used in [ref. 40]), in which the same animals are passively exposed to the same set of stimuli, but in which rewards were not provided. That is, there is no task and thus no outcome category per trial.

  If correlations accompany task processing, we would expect them to be weaker than for correct-choice outcome trials, in which the task has been performed successfully. This is suggested in the new Supplementary Figure S1: Triple passive correlations on such ensembles are indistinguishable from incorrect trials; and are significantly lower than correct ones. Further details can be found in the in the Supplementary Material, new Supplementary Figure S1 and caption and in the main manuscript, lines 136-141.

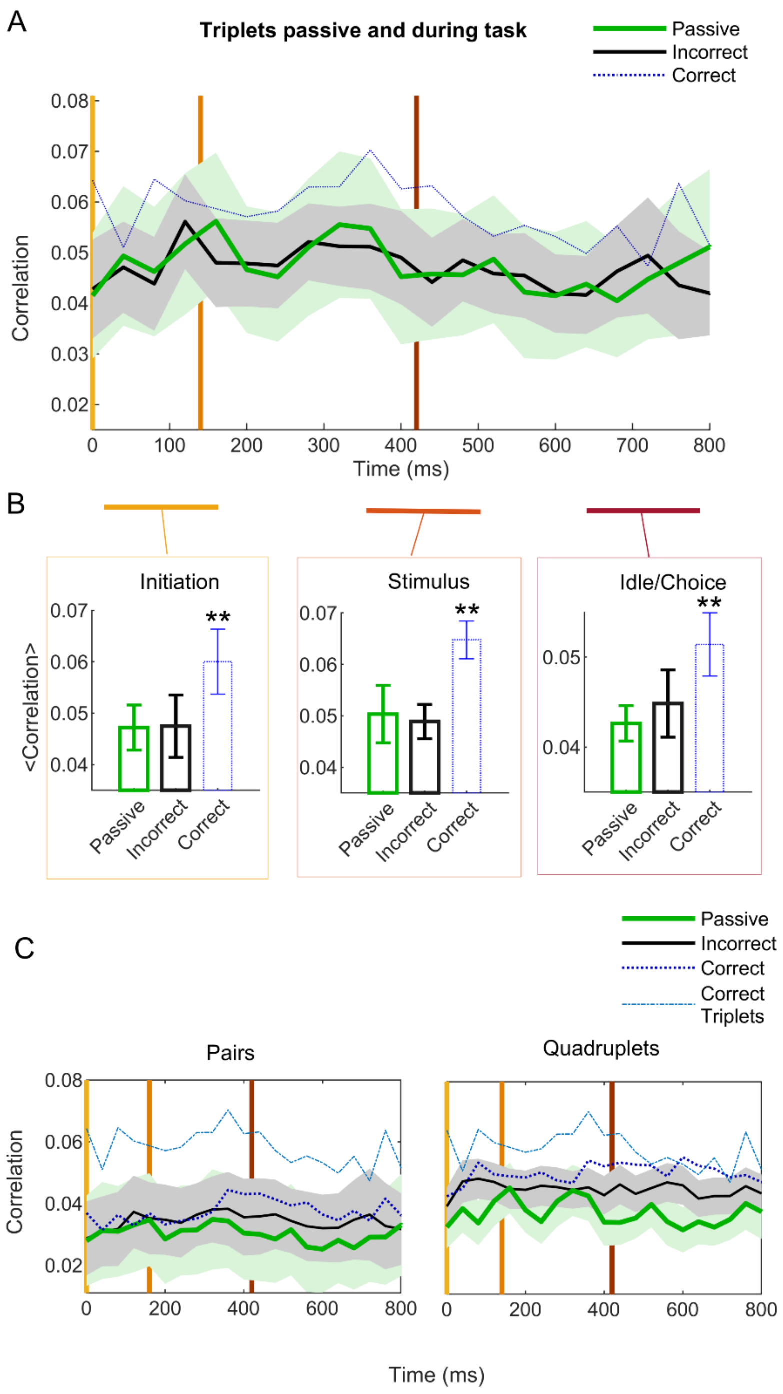

Figure S1

- **New analysis 2: Correlations clarification and new Supplementary Figure S5.** As the reviewer indicates, Figure 1B shows that the difference between the absolute value of 'noise' correlation coefficients for correct and incorrect outcomes seems roughly steady during the trial. This figure plays the role of an illustrative, opening example using the largest ensembles, as discussed before and below in this letter in ***RC5*** and ***RC6***.

  On the other hand, as the reviewer says, it is known that single-units rates encode information on the immediately previous outcome and choice before stimulus presentation ([ref. 40], see also ***RC1***).

  In this manuscript, we propose that, in addition to this, *signed*, especially infrequent negative third-order correlations further modulate decoding; and do so beyond this first-order Markov model.

This is suggested in Figure 6: For both trial types (termed here 'predictable' and 'unpredictable') negative correlations coefficients are roughly 1/3 of the total (Fig 6C). However, unlike positive ones, they are predictability-dependent: During unpredictable trials, negative correlations are significantly larger for correct-choice outcomes than for incorrect ones (Fig 6A, left). In contrast, during predictable trials (Fig 6A, right), this distinction is inverted during early trial stages (Fig 6A, right, 'Initiation' inset) and then vanishes.

We thus propose that, only for predictable trials, the detrimental effect of negative correlations in decoding is negligible, since in such trials the information on the optimal decision is already available during the trial initiation. We created a new figure (Supplementary Material, new Figure S5) to clarify this effect.

In short, for each correlation coefficient, we derived a simple surrogate index, termed here the *differential correlation coefficient* $\Delta\delta = \delta^{+} - \delta^{-}$ (Supplementary Material, new equation (S18)) which is calculated separately for predictable and unpredictable trials, and for each one of the trial periods (initiation, stimulus, choice). The quantity δ^{+} (likewise δ^{-}) consists of the difference in positive (negative) correlation between correct and incorrect trials, where correlation coefficients are aggregated during a specific time period (Supplementary Material, new equations (S18) and (S19)).

Thus, large $\Delta\delta$ suggest that positive correlations express more strongly the difference between correct and incorrect trials than negative ones collectively during a specific time period. According to our hypothesis, we expect $\Delta\delta_{predictable} > \Delta\delta_{unpredictable}$ at least during the trial initiation period to foster decoding, when no information on the upcoming stimulus is available.

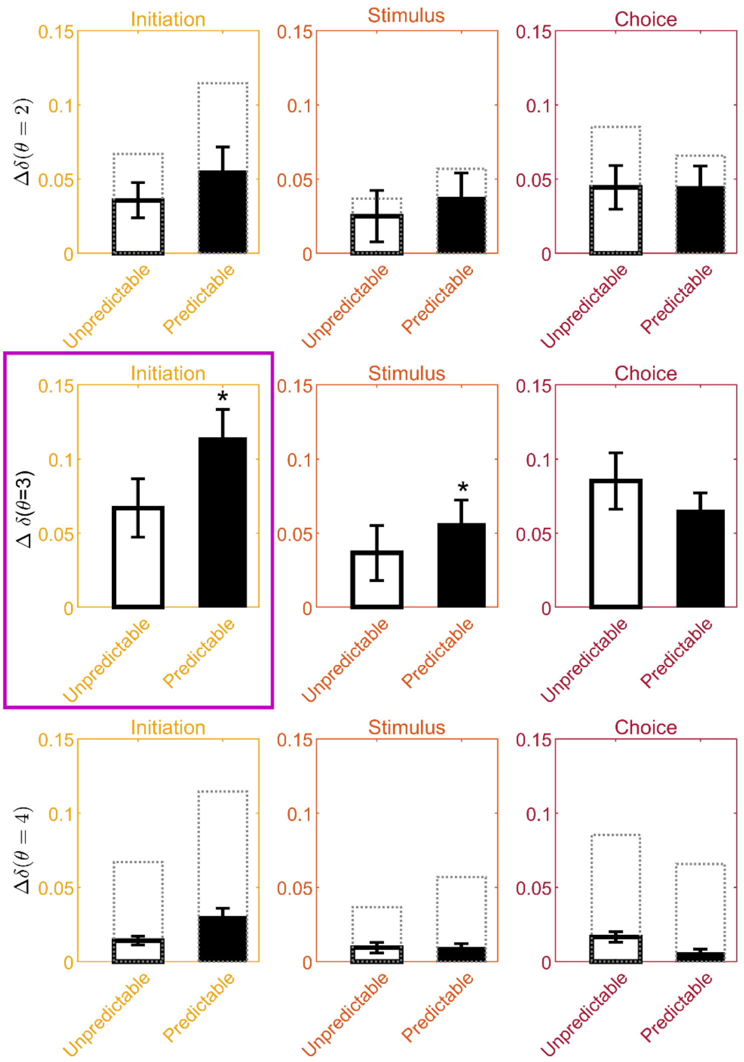

Figure S5

This is shown in the new Supplementary Figure S5 (see figure above, dotted lines indicate third-order correlations for comparison, middle panels, $\theta = 3$). Interestingly, this effect does not reach significance for pairwise correlations; and vanishes for quadruple-wise correlations (dotted lines show the third-order correlations for comparison), due to their weaker and less outcome-specific *differential* correlations (top and bottom rows).

Please note also that this mechanism is not fully explained by a first-order, linear Markov chain in consecutive trials. The reason is that, when this information is subtracted by linear regression (see Methods, equation (2)), correlation coefficients of the residuals show the same trend. This is shown in Figure 7 in the revised manuscript (formerly in the Supplementary Material section, we have also produced a new Supplementary Figure S3 to further support this from a decoding angle, see **New analysis 3** discussed below).

The correlations effect is discussed on lines 279-292 in the main manuscript and now further discussed on new lines/paragraphs in the main manuscript, e.g., on lines 293-303, 304-312, 394-397.

The description and analysis of the new index are shown in the Supplementary Material, lines 152-164 (new equations (S18), (S19)) and in the new Supplementary Figure S5 and its caption.

- **New analysis 3 (new Supplementary Figure S3).** Along these lines, and to further clarify the effect of high-order interactions, we checked if the decoding effect shown in Figure 4 is still present when the residuals are used for decoding instead of the original data. That is, if after optimally regressing the current trial with the immediately previous one, whether it is still possible to decode correct trials with a higher performance than incorrect ones.

  This would suggest that linear information transferred between consecutive trials, although fundamental, it is not the only source of information that is being used for decoding. In other words, predictions drawn from the first-order Markov hypothesis can be improved to some extent. This analysis is shown in the Supplementary material, new Figure S3, shown also below.

  In short, dotted lines correspond to the original data, in which the previous trials have not been regressed. Orange triangles are bootstraps constructed in the same way as in Fig. 4. The effect is weaker as expected, but even on the residuals, the correct outcome trials are still better decoded. Like in Figure 4, the left panel shows the causally cross-validated decoding error (DE), whilst the right panel shows the trajectory divergence index (DE).

  Details can be found in the Supplementary Material, Figure S3 and its caption and in the main manuscript, lines 225-234.

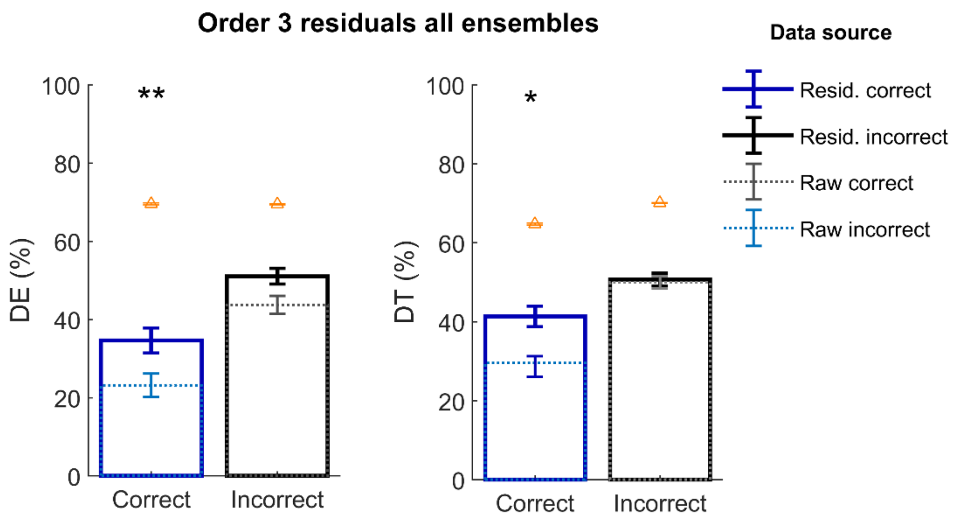

Figure S3

II. Specific Concerns:

Reviewer comment 4 (RC4): *"Line 101: "increases during correct choices". No, even taking the authors at face value it does not increase during the course of correct choices -- it is higher from the outset of the trial (cf. Fig. 1B)."*

Thank you, the imprecision has been clarified: "Shared variability among triplets is higher for correct-choice outcomes" in the main manuscript, lines 101-102.

Reviewer comment 5 (RC5): *"Lines 123-124: what data values are these tests computed for? The mean computed over the course of the trial? Why is there no correction for multiple comparisons given that the test is being computed for pairs, triplets, quadruplets... constructed dipping from the same data? By the same token, one could test ever-increasing correlation orders (quintuplets...) (although with decreasing statistical power, so significance would be less achievable) until a significant result was found."*

"Lines 125-129: again, these post-hoc comparisons are being made multiple times. The authors are handpicking a series of time windows and running the same analysis. Sometimes, the result is borderline significant (p = 0.42). Are these really "highly significant" effects?"

Thanks for pointing this out, we have clarified the text in the revised version as follows:

- Significances in Fig 1B, D compare correlation coefficients of the same order computed from two classes of trials (are obtained from different datasets). Note that trials are already averaged out in the definition of 'noise' correlation (equation (1)). Thus, there is an array of distinct 'noise' correlation coefficients per time bin, and specifically in Figure 1 we take their average and consider the bins of interest (either the entire trial or a specific period), to compare two types of trials. It is thus a pairwise comparison for a fixed correlation order.

  Now, for clarity, we report in addition a MANOVA in which we do not average the coefficients; thus, bins are multivariate dimensions, and groups are the two types of trials. That is, each correlation coefficient is characterized by a vector; this is now reported in the main manuscript, line 125-126. We also reported the significance of mean coefficients during the entire trial duration in lines 126-127. Averaging over time instead yields to similar conclusions. In addition, it would be interesting to report a flipped MANOVA in which dimensions are the distinct coefficients, but we would need a much longer trial to reliably compute covariance matrices. Normality is checked in every case of the manuscript using conservative Lilliefors tests to apply the correct test, as indicated in lines 504-505.

  In all places where multiple comparisons were performed in the manuscript (e.g., in 1D and in Figure 2C in which we compare four types of trials, see note below) the post-hoc *p*-values shown are Bonferroni-corrected as the reviewer indicates (e.g., line 179-180, please see ***RC6*** below).

- However, there is a comparison between orders 2, 3, 4 dipped from the same data, this is the one performed in Fig 1C. We omitted the ANOVA and *p*-values because their significance was very salient; now this is reported in the main manuscript, lines 132-135, the significances in the figure refer to Bonferroni-corrected post-hoc comparisons.

  We note that neither quadruplets nor pairwise correlations express significant differences between correct and incorrect trials regardless of the distinct number of coefficients (Fig 1B, C). Thus, we did not appreciate an effect on the number of coefficients (at least 124 distinct ones for order 2).

  Quintuplets can be computed at a high computational cost, but we agree with the reviewer that they would be biased here, since most of the ensembles are too small (we would need to use only ensembles of 6+ units otherwise we would have at most a single 5th order coefficient in which neurons are different), and hence we cannot compare them in fairness with the rest of the orders (they are also very small, see ***RC8*** below). This is also succinctly discussed in the main manuscript, lines 384-386 (and already indicated in ***RC3, new analysis 2***).

- The periods of behavioural interest also enable us to compare our work with ref. [40], in addition please see ***RC2*** for the specific interest of the trial initiation and choice periods. Thank you for spotting the incorrect ‘highly’, it has been removed.

Reviewer comment 6 (RC6): *“What makes the initial data (Fig. 1C) different from later plots (Fig. 2C) where the correlation values shown are different? I can't understand why the value for (correct trials, triplets) in those two panels is different. Did different ensembles/units go into each of the calculations (out of what is, overall, quite a small data set -- apparently 82 units at most)?”*

We clarified now that Figure 2C shows only an individual ensemble as an example; this is indicated in lines 167-168 and in the figure caption. This ensemble was selected since the correlation was particularly high and hence the visual representation of correct choices was particularly stable, in order to showcase better the forthcoming analyses with all data (likewise, Figure 3 shows a specific case for one of the smallest ensembles). Statistical analyses using all ensembles are shown in Figure 4. We in added several clarifications in Figures 2 and 3 captions (lines 687-688, 707) and a note in the main manuscript, line 168.

Reviewer comment 7 (RC7): *“Decoder/discriminant analysis (Fig. 2): Fig. 2B finally shows that there's something different in neuronal activity between correct and incorrect trials and that this is picked up by a population analysis […].*
*“Lines 209-214 and Fig. 4 top panels: I find this analysis really puzzling. The decoding error for the 4th order decoder on correct trials is higher than for the 3rd order decoder. If one includes terms with 4th order interactions in addition to those up to 3rd order, shouldn't the decoding error have a lower bound equal to the error for the decoder up to 3rd order? This is because, as the coefficients for 4th order terms go to zero, the result should match the 3rd order decoder -- there is no way that including more terms can make it worse. I'm willing to believe that adding terms to 4th order may not improve decoding much, but the plot clearly shows a value that is *worse*. NB this is presented as a decoding error measure, not a quality of fit or AIC measure.”*

Thank you, we will explain now these queries.

- The subtle increase the cross-validated error observed in the inset of Fig. 4 for orders 4+ is a typical case of slight over-fitting arising in kernel machines using this specific kernel-reproducing Hilbert space. For instance, a U-shaped curve in the validation error (bias-variance trade-off-like) can be appreciated in our previous papers, e.g., Figure 3 in ref [37], Figure 3 in ref [55] etc. or in the machine learning literature (refs. [60], [82]).

  Please note that we always show causally cross-validated error (from past to future trials), not adjustment of a classifier over a dataset (lines 626-636 in the main manuscript, Methods). In this setting, higher-dimensional spaces tend to generalize worse eventually, since once fixed the regularization constant, order is the only effective hyper-parameter of this classifier. This overfitting effect is equivalent to varying the value of the radial basis kernel width in this classifier or in an SVM-like one (e.g., in Fig. 7 in ref. [82]).

- As the reviewers indicates, AIC/BIC enable us to penalize the excess of regressors; and are a common index in ARMAX filters to identify the minimal set of lags even on training sets (Balaguer-Ballester et al., 2002, Ecological Modelling).

  Here, the optimization is based on equation (7) maximization of a classifier, and the decoding space is not directly accessible due to the high dimensionality. This prevents a systematic elimination of dimensions. If we could systematically remove the dimensions in turn like in a regression model, or more broadly using some form of recursive feature elimination, it would yield to the result indicated by the reviewer. That is, extra dimensions containing quadruplet interactions or beyond do not contribute significantly to decrease the out-of-sample error and can be discarded. In short, the cross-validation we conducted also penalizes the excess of dimensions, parametrized by the interactions order. We added a brief note in the main manuscript, line 224 we are very happy to expand further if requested.

Reviewer comment 8 (RC8): *"Lines 278-279: "the ensemble state during correct-choice trials is robust when the optimal choice can be predicted from the previous trial outcome; it temporarily destabilizes otherwise." This sounds believable and can be deduced from the decoder analysis. But that OFC can host a trial history-dependent population signal is not new, as the authors themselves point out later: "suggests that the cognitive map of the task-space provided by OFC units [63] enable them to encode a compact combination of past-trial state variables, which can predict the upcoming decision [40]." In other words: OFC contains a signal that says "I did it wrong last time, and that means the next stimulus is like the last one." But the authors' approach appears to fall short in identifying the substrate of the signal: the triplet correlation analysis is not convincing at least as presented, and correlation statistics for orders > 3 may well be underpowered given the (apparently) limited dataset."*

Thank you very much for the positive analysis. This has been addressed in the ***RC3*** section **new analysis 3** in this letter, e.g., in the Supplementary Material, new Figure S3 and in the main manuscript lines 225-229, 304-312 and in Figure 7 and its caption.

Please see also ***RC2*** and ***RC5*** for discussions on correlations power and causality.

As the reviewer indicates, we cannot compute reliable correlations beyond quadruplets in this dataset, nevertheless, they are very small here ($<10^{-3}$). However, neglecting such correlations for decoding is only a hypothesis still to be tested on larger OFC ensembles and with the support of information-theoretic-based methods providing proxies for correlation coefficients (due to the very high computational cost of a direct computation beyond triplets).

On this topic, we have added discussion paragraphs in the main manuscript, lines 381-397 already mentioned in ***RC3, new analysis 2*** and in ***RC5.*** We also added five new related references in the main manuscript, numbers [66-68], [71], [74-75].

We clarified that we also provide the three animals datasets for public use in the main manuscript, lines 659-660.

Reviewer comment 9 (RC9): **Minor:**

L. 189: "W < 57, p < 0.031" Are these really less than, not equal to? If so, why wasn't it possible to quote the actual value?

"lowly" is misused and there are other typos/mistakes -- the manuscript could do with an edit.

L. 366-369: two or three rats?

L. 626: "Same as in A" - B.

We now report individual test values for CE and DIV on the full dataset in the main manuscript, line 211.
We changed "lowly" for "weakly" as advised. We also corrected the mistaken page numbers/year in the main manuscript, references 3 and 73 and other minor edits in references. Thank you very much for spotting these errors, is three animals, corrected on line 419.

Reviewer 2

The extremely positive and complimentary statements from the reviewer are much appreciated.

RC 1. *"The current paper entitled "Reliable Representation of Foreseeable Choice Outcomes in Orbitofrontal Cortex Triplet-wise Interactions" constitutes, in my opinion, a very important contribution to the field of computational neuroscience as it is a remarkable study about the impact of triplet correlations on reliable representation of foreseeable outcomes in the orbitofrontal cortex (OFC).*

The current research is extremely important as it shows the relevance of triplets' correlations on perception and cognition. Their results prove that correlations among triplets are higher during correct choices with respect to incorrect ones, and that this is sustained during the entire trial, moreover this effect is not observed for pairwise nor for higher than third-order correlations.

Moreover, the current study provides invaluable proof that we cannot simplify the functioning of the neuronal code to naive models of pairwise correlations since they can lead us to completely erroneous interpretations when one wants to investigate how information is processed in the brain. Third-order correlations are therefore a very relevant issue on information transmission in the neuronal cortex. More specifically, their results provide evidence that correct choice predictability and hence the optimal behavioral strategy is encoded in metastable states temporarily assembled by complex, third-order lateral OFC (lOFC) constellations.

The current study constitutes a major contribution to the field. The results are carefully analyzed and the work is formally speaking very well presented. Overall, I think that this is paper is a very important one and it should be published in PLOS Computational Biology.

However, I have some very minor suggestions:

-Please notice that volume and pages are missing in Ref [3]: Proc Natl Acad Sci U S A. 2017 114(12):E2494-E2503.

-Please correct the year of reference [69] as it is from 2013 (not 2012). Physica A 392 (2013) 3066-3086 and please also cite a more recent paper where the mentioned formalism has been extended: L Montangie, F Montani, "Higher-order correlations in common input shapes the output spiking activity of a neural population" Physica A: Statistical Mechanics and its Applications 471 (2017) 845-861."

Thank you very much for spotting these errors and for the updated references, now correct and added.

-***RC 2***. *"At theoretical level a recent paper has shown that third order correlations can induce synergy/redundancy states in the information-theoretic sense, thus please cite the following paper: "Effect of interacting second- and third-order stimulus-dependent correlations on population-coding asymmetries" by Lisandro Montangie and Fernando Montani Phys. Rev. E 94 (2016) 042303.*

-As the discrimination among choice outcomes vanishes for weaker higher than third order interactions it would be of interest if the author could also discuss a possible role of higher order silences (in similar fashion to Hideaki Shimazaki et al Scientific Reports 5 (2015) 9821)."

Thank you very much for the reviewer's specialized input. These papers are intriguing, directly relevant to our results and enrich the depth of our discussion. We incorporated new paragraphs to main manuscript, lines 381 to 397 and we added the new references, numbers [66-68], [71], [74-75].
