# Supplementary material for: Representation of foreseeable choice outcomes in orbitofrontal cortex triplet-wise interactions
Source: PLoS Comput Biol. 2020 Jun 24;16(6):e1007862. doi: 10.1371/journal.pcbi.1007862 (PMC7313741; doi:10.1371/journal.pcbi.1007862)
Supplement: Attachment — Submitted filename: Rev_2_Reviewers_Responses_Letter_Balaguer_B_et_al.pdf [file pcbi.1007862.s008.pdf]

Reviewer 1 comment:” *The authors have thoroughly revised the paper and have convinced me of the interest of their analysis. This is a nice follow-up to their earlier studies and the notion (and finding) that triplet interactions may contain a code for choice outcomes is intriguing.*

That said, there are one or two places where the importance of the findings (which remain largely correlational, in terms of the effects of triplet interactions upon the animal's behavior) is still somewhat overspun. In the Author Summary, the authors could tone down the proposal that "coordinated responses of up to three neurons in the OFC are fundamental for the capacity of the animal to take the optimal decision".

The manuscript also has a few typos so I would suggest an extra edit. A non-exhaustive list:
"orbitofrotal" (l. 45)
"analysis seem consistent" (l. 142) (should be analyses, or seems)
"demonstrates how that third-order correlations" (l. 383)
"enables" should presumably be "ensembles" (l. 397)

The very positive and complimentary statements from the reviewer are much appreciated. Thank you very much for the detailed analysis of the manuscript and for the useful input provided. Our apologies for these typos, we have edited these lines and the rest of the imprecisions and typos throughout the manuscript. We have also added a very recent reference. As the reviewer indicates, the concluding claim in the Author’ Summary was an overstatement, we have toned it down, consistently with the rest of the manuscript.

Please find these and other minor editions along these lines highlighted in the revised version, both in the main text and in the supplementary material. We would be delighted to provide the large datasets by request and/or via any public repository suggested by the Plos Computational Biology team.

Reviewer 2 comment:” *The improved version of the manuscript is very solid and interesting.*

I recommend its publication.”

We would like to thank the reviewer very much for the specialized and useful input provided.
